# Pooled prevalence, spatial variation and associated factors of HIV testing uptake among multiple sexual partners in Sub Saharan Africa: Spatial and multilevel analysis

**DOI:** 10.1371/journal.pone.0306770

**Published:** 2024-07-11

**Authors:** Emebet Birhanu Lealem, Ejigu Gebeye Zeleke, Betelhem Abebe Andargie, Alemakef Wagnew

**Affiliations:** Department of Epidemiology and Biostatistics, Institute of Public Health, College of Medicine and Health Sciences, University of Gondar, Gondar, Ethiopia; United States Agency for International Development (USAID), NIGERIA

## Abstract

**Background:**

Uptake of HIV testing is vital for the early diagnosis of HIV infection and initiation of treatment, which are used to eliminate the disease’s progression and reduce HIV-related mortality. Even if determining HIV testing is imperative to prevent HIV/AIDS among multiple sexual partners who are at higher risk of sexually transmitted infections, most of the countries in Sub Saharan Africa did not fulfil the global targets of UNAIDS. Moreover there is a paucity of literature on spatial variation and factors associated with HIV testing among high-risk groups in SSA. This study aimed to assess the pooled prevalence, spatial variation and determinants of HIV testing uptake among multiple sexual partners in Sub Saharan Africa.

**Methods:**

The Demographic and Health Surveys data conducted between 2011 and 2021 in 30 Sub-Saharan Africa countries was used to analyze total weighted sample of 56,210 multiple sexual partners. Exploratory spatial data analysis, with countries as the unit of analysis was conducted using ArcGIS V10.7.1 and Sat Scan V 10.1 soft wares. A multilevel binary logistic regression model was used to identify the factors associated with the HIV testing uptake. The Adjusted odds Ratio with a 95% confidence interval was reported to declare the strength of association and their statistical significance.

**Results:**

The spatial patterns of HIV testing uptake were found to be non-random. Primary clusters were identified around western and central sub- regions. Multiple sexual partners who were ever married, those attended primary level and above education, those from rich wealth status, aged above 24 years, having good HIV related knowledge, and exposed to media were positive association with HIV testing uptake. However, being male, having working status and living in rural area were negatively associated with HIV testing uptake. At the community-level, multiple sexual partners from communities in Eastern and southern sub regions, countries with upper middle income and countries with the survey year after 2014 were more likely to utilize HIV testing services compared with their counterparts.

**Conclusion:**

In this study, the pooled prevalence of the HIV testing uptake among multiple sexual partners was found to be lower than the universal target and showed differences in HIV testing uptake across Sub-Saharan Africa region. Both individual and community-level factors affected HIV testing uptake among multiple sexual partners. Stakeholders should implement interventions to help increase the uptake of HIV testing among those risky groups in this region.

## Introduction

Multiple sexual partnership (MSP) refers to being with more than one sexual partners over a period of time [1, 2]. It is a significant public health focal area in many countries, particularly in Sub-Saharan Africa (SSA) [2, 3], by raising sexually transmitted infections (STIs) [4]. People who have a number of sexual partners increase the possibility of STIs transmission to their faithful spouses which is likely driver of the spread of human immunodeficiency virus (HIV) [5].

HIV/AIDS pandemic has continued to be a serious universal health issue, Sub-Saharan Africa remains the most affected region more than any other regions in the world [6, 7]. As shown by USAIDs reports in 2022, sub-Saharan Africa accounted for 51% of all new HIV infection [8, 9].

According to the Joint United Nations Program on HIV/AIDS (UNAIDS) national fact sheet 2021, 1.5 million persons acquired HIV, and approximately 650, 000 peoples died due to HIV-related causes. HIV related deaths have also been reduced by over 60% as a result of a successful HIV testing, treatment and support programs [10].

Uptake of HIV testing is very essential in order to prevent the spread of the disease and lower HIV-related mortality, early detection of HIV infection and the beginning of treatment [11]. But, many HIV infections stayed undetected, according to the World health organization estimate, only 54% in 2015 and 85% in 2021 of people with HIV are aware of their HIV status [12, 13]. Some other studies conducted in China and Georgia reported that from multiple sex partners 12.4% [14, 15] and 66.9%, [16], 15.5% in Germany [17] and 43.5% in Portugal [18] had evidently ever tested. Africa has a range in HIV testing participation of 10.0% in Burkina Faso [19] to 69.9% in Malawi [20, 21]. According to the 2017 UNAIDS report, SSA is home to a sizeable share among the 9.4 million people living with HIV (PLHIV) and those who are unaware of their status [22, 23].

Evidence suggests that postponing HIV testing causes late diagnosis, which is hazardous for the individual as well as the general population [11]. In other words, people who wait too long to get a diagnosis typically have a higher mortality risk, a worse prognosis, and need more expensive medical care. By declining to adopt the behavioral patterns and preventative measures required to stop further illness transmission, those who refuse to get tested contribute to the disease’s pandemic nature [24].

Age, place of residence, gender, marital status, and socioeconomic position are among the factors that influence and facilitate the uptake of HIV testing, according to prior research [10]. Contrarily, perceived low risk of HIV infection caused by low community literacy level, fear of stigma or perceived psychological burden of living with HIV, low level of comprehensive knowledge towards HIV/AIDS, and lack of access to media are reported as being more or less barriers to the uptake of HIV testing [10, 25, 26].

In 2014, UNAIDS approved the 90-90-90 strategic framework to eliminate HIV/AIDS [27], which stimulates that in order to decrease and ultimately eradicate new infections in key populations, voluntary counseling and testing (VCT) should be increased so that 90% of PLWHA know their status by 2020 and later expanded to be 95-95-95 by 2030 [27, 28]. The Sustainable Development Goals (SDGs) and the Centers for Disease Control and Prevention (CDC), two linked organizations, have each issued different suggestions addressing the HIV epidemic. Access to HIV prevention and treatment is a concern addressed by SDG 3. While the CDC recommends that individuals with certain risk factors undergo testing at least once a year and that everyone between the ages of 13 and 64 undergo testing at least once as part of routine healthcare [29–31].

In line with this program, after the declaration of national HIV/AIDS policy; different countries started variety of comprehensive intervention programs which is integrated with health education and focus on voluntary counseling and testing (VCT) [32–34].This help to enhance knowledge of HIV status, improve uptake of antiretroviral treatment (ART), and prevent transmission of HIV that may lead to reduce HIV transmission potential and improve HIV-related morbidity and mortality that increase life expectancy [35–37]. However, only 12 sub-Saharan African (SSA) countries reached the first 90% target (90% of people living with HIV to know their status) [38]. Due to this, different studies should be conducted in the area.

Even if determining HIV testing is imperative to prevent HIV/AIDS and to increase uptake among multiple sexual partners who are at higher risk of infection, its pooled prevalence, factors affecting and also the spatial variation of this HIV/AIDS prevention strategy is not assessed yet. Therefore this study aims to assess the spatial variation, and associated factors of HIV testing uptake among multiple sexual partners in sub Saharan Africa using recent demographic health surveys.

## Methods and materials

### Data source

Sub-Saharan Africa is made up of 48 countries. Among this seven countries (Cape Verde, Equatorial Guinea, Guinea-Bissau, Mauritius, Seychelles, South Sudan, and Somalia) have no DHS data or not publicly available. Among the remaining 41 counties, countries such as Mauritania, Nigeria and Tanzania, have no record of outcome variable and in the other hand, data sets of Central Africa Republic (DHSreport1994/95), Eswatinia (DHS report 2006/07), Sao Tome Principe (DHS report 2008/09), Burkina Faso (DHS report 2010), and Sudan (DHS report 1989–90) are not recent enough (in 2011–2021). As well as Sub-Saharan Country (Congo Republic) was excluded from the available sample data sets since its coordinate data sets are not publicly available. After excluding countries that had no DHS datasets after 2011 and countries where the DHS dataset was not publicly available or no observations for the study participants or outcome variable and also no coordinate data, 30 countries were extracted in this study.

### Study design and period

The Demographic and Health Surveys (DHS) data were used; it is a cross-sectional nationally representative survey conducted in five years to produce updated health and health-related variables. The data used for this study was recent standardized DHS data from available nations in Sub-Saharan Africa. These DHS data were representative of each country at a specific point in time with in the interval of ten years (2011-2021GC) [39–41].

### Study setting

The research was based on a DHS survey among countries in Sub-Saharan Africa (SSA). The Sub-Saharan is the territory of Africa that lies on southern part of the Sahara. It consists of around forty eight countries [42] and divided into four enormous and separate sections: Eastern Africa (Burundi, Comoros, Ethiopia, Eritrea, Kenya, Malawi, Mozambique, Madagascar, Mauritius, Rwanda, Seychelles, South Sudan, Sudan, Somalia, Tanzania, Uganda, Zambia, Zimbabwe), Central Africa (Angola, Cameroon, Central African Republic, Chad, Congo, the Democratic Republic of the Congo, Equatorial Guinea and Gabon, Sao Tome and Principe), Western Africa (Benin, Burkina Faso, Cape Verde, Ivory Coast, Gambia, Ghana, Guinea, Guinea- Bissau, Liberia, Mali, Mauritania, Nigeria, Niger, Senegal, Sierra Leone, Togo) and Southern Africa (Botswana, Eswatinia, Lesotho, Namibia, South Africa). The study area cover 9.4 million square miles and have a total population of 1.18 billion inhabitants [43].The datasets can be found on the DHS website, www.dhsprogram.com [41].

### Source population and sampling procedures

All women and men with multiple sexual partnership in sub-Saharan African (SSA) countries. Generally, all selected national surveys used the most recent census frame. DHS samples are often stratified by geographic region and within each region, by urban/rural areas. Clustered two-stage probability samples generated from an existing sample frame was used. For the first round of sampling, Enumeration Areas (EAs) were used as sampling units. The second stage of sampling was selecting fixed number of Households (HHs); around 25–30 households per PSU in several EAs. Following the listing of households, equal probability systematic sampling is used to select a specified number of households in the designated cluster. Each DHS report on the Measure DHS website included a comprehensive sampling technique (www.dhsprogram.com). Weighted values was computed using Individual women’s records (IR) and individual men’s records (MR) DHS datasets to restore the representativeness of the sample data. Finally, this study was comprise a total weighted sample of 56,210 multiple sexual partners from selected nations [41] (Table 1).

**Table 1:** Sample size determination of HIV testing uptake and its associated factors among multiple sexual partners in each Sub-Saharan Africa: Based on 2011–2021 DHS.

Regions	Country	survey year	Un-weighted Sample size	Weighted Sample size
**Eastern Africa countries**	Burundi	2016/17	255	267
	Comoros	2012	402	403
	Ethiopia	2016	565	504
	Kenya	2014	18,042	18,324
	Malawi	2015/16	1295	1266
	Madagascar	2021	2601	2598
	Mozambique	2011	2606	2303
	Rwanda	2014/15	509	540
	Uganda	2016	1559	1527
	Zambia	2018	1980	2041
	Zimbabwe	2015	1402	1366
**Central Africa countries**	Angola	2015/16	1334	1294
	Cameroon	2018	2253	2167
	Chad	2014/15	1152	1135
	DR Congo	2013/14	2494	2462
	Gabon	2012	2454	2491
**Western Africa countries**	Benin	2017/18	1891	1903
	Cote d’ivoir	2011/12	1749	1848
	Gambia	2019/20	613	578
	Ghana	2014	709	758
	Guinea	2018	703	697
	Liberia	2019/20	1569	1566
	Mali	2018	823	892
	Niger	2012	643	680
	Senegal	2019/20	727	623
	Sierra Leone	2019	2109	2130
	Togo	2013/14	858	852
**Southern Africa countries**	Lesotho	2014	1240	1281
	Namibia	2013	751	765
	South Africa	2016	958	949
**Total Sample size**			56,246	56,210

### Study variables

#### Outcome variables

The outcome variable is HIV testing uptake which is a single direct question asking whether the respondent has ever been tested for HIV or not (the response was Yes/No). The outcome variable was recoded as “tested for HIV = 1” and “not tested for HIV = 0”.

#### Independent variables

Individual and community-level independent variables were studied. The individual-level factors were Age, Sex, Marital status, Educational status, Occupational status, Wealth status, Media exposure, Early sexual initiation, Risky behavior, Level of knowledge about HIV/AIDS. The place of residence, Sub- regions in Sub-Saharan Africa, Survey year and Country income level were the community-level factors.

### Operational definitions

**Multiple sexual partnership (MSP):** is defined as having two or more sexual partners during the 12 months before the survey. It was coded as "1" when the participant had two or more sexual partners and "0" when there was one sexual partner [44].

**Sub-region of Sub-Saharan Africa**- The sub-region of sub-Saharan Africa (Eastern Africa, Central Africa, Western Africa, and South Africa) in which countries are found.

**Country income level:** The country’s income status is based on the World Bank list of economies classification in 2011–2021 (according to their survey year) [45].

**HIV knowledge**: It was generated based on three questions related to HIV prevention and three questions related to the modes of HIV transmission and graded as low (if a respondent answered ≤3 questions correctly), high (if a respondents answered 4–5 questions correctly) or comprehensive (if a respondent answered all the 6 questions correctly). Therefore, the knowledge can be generally considered as “low knowledge” if the respondent answered ≤3 questions correctly and “high knowledge” if a respondents answered 4 and above questions correctly [46].

**Early sexual initiation:** is the experience of first intercourse before 18 years of age [47].

**Risky sexual behaviors**: Assessed based on the four questions; having had STI in last 12 months, genital sore/ulcer in last 12 months, and genital discharge in last 12 months. These were combined into an index of risky sexual behavior with two categories: namely “No risk” if the response is no for all questions and “Have risk” if the response is yes for one or more of five questions [46].

**Mass media exposure**- is defined as the individual who had at least been exposed to one media, television, radio, or newspaper. It was coded as “0” for low, and “1” for high media exposure [10].

### Data quality control

Data quality of the data was confirmed using standardized data collection tool and procedures. Data from the DHS can be compared between countries. Both missing values and non-applicable data are clearly defined in the DHS data guideline, and they was handled accordingly. Each country’s data consistency was reviewed before all 30 countries’ data and both Individual women’s record (IR) and individual men’s record (MR) was appended. The supervisor and investigator was review the data for consistency and completeness.

### Data management and analysis

The data from the DHS dataset was downloaded in STATA format. The data was cleaned, categorized, converted and also checked before the next step of data management. In order to prevent over powering country surveys with smaller sample sizes by those with large sample sizes the data was adjusted with the sample weights such that each country would carry equal weight in the combined dataset. The STATA version 17 software was used to data handling and constructing descriptive and analytic statistics.

#### Spatial analysis

*Spatial distribution*. Spatial data analysis was done using ArcGIS software version 10.7.1 to identify geographic variations of HIV Testing uptake among multiple sexual partners in 30 sub-Saharan countries. The spatial analysis with the countries as the unit of analysis in a geographic coordinate polygon shape file of SSA [48]. The CSA (Central Statistical Agency) database was provide the African district delineation shape file. The coordinate points in shape file format was used for each country from the DHS office upon request. The shape file has a standard World Geodetic System 1984 (WGS84) and Universal Transverse Mercator (UTM) Zone 37°N which forms its angular units in degrees and Greenwich as the prime meridian [40].

*Spatial autocorrelation*. Spatial autocorrelation analysis was used for evaluating whether the patterns of HIV testing uptake among multiple sexual partners is clustered, randomly or dispersed distributed by means of Global Moran’s I spatial analysis. Global Moran’s I statistic measures produce a single output value whose ranges were between− 1 to+ 1. If its value closes to − 1 which indicates dispersed HIV testing uptake, whereas when it is closest to + 1 indicted clustered HIV testing uptake and the I value is 0 which indicates randomly distributed HIV testing uptake. The Moran’s I (P-value < 0.05) indicates the presence of spatial autocorrelation. Z-score was computed to determine the statistical significance of clustering, and the p-value was computed for the significance [49]. The global Moran’s I statistic was used to assess the overall spatial autocorrelation, will not identify the specific locations of the clusters.

*Cluster and outlier analysis*. In order to solve the concern of global Moran’s I statistic, the local indicator of spatial association (LISA) was done. In addition to the local spatial autocorrelation it used to determination of outlier clusters. Its cluster maps showed the significant locations in four color-coded categories: low–low, high–high, low–high, and high–low. The terms low and high will be defined relative to the overall mean of the indicators. The high–high and low–low locations (positive local spatial autocorrelation) are referred to as spatial clusters, while low–high and high–low locations (negative local spatial autocorrelation) are referred to as spatial outliers [50].

*Hotspot analysis*. The local Getis-Ord statistic (Gi*), also known as hotspot analysis, was used to provide further evidence that point out the intensity and stability of core hotspot or cold spot clusters of HIV testing uptake in the entire Sub-Saharan Africa [51]. The Getis-Ord Gi* statistic serves as an indicator for local autocorrelation, which measures how spatial autocorrelation show a discrepancy locally over the study location and then computes a statistic for each point data [52].

The degree of clustering and its statistical significance is estimated based on a confidence level, according to the Z-scores. If the Z (Gi*) score is positive and significant, it shows that one district and its neighboring regions have a relatively high frequency of HIV testing uptake, which is a hotspot (a spatial cluster of high data values); on the contrary, if the Z (Gi*) score is negative and significant, it indicates a cold-spot (a spatial cluster of low data values) [53].

*Spatial interpolation*. Implementation of services should determine the impact of a particular event in exceeding difficulty in time- and resources for their both effectiveness and efficiency. Since, it is the method by which part of a particular area can be anticipated using observed data, it plays a great role in solving this challenge. The spatial interpolation technique was used to predict HIV testing uptake in the un-sampled areas in the country based on sampled enumeration areas. There are different deterministic and geo-statistical interpolation methods. In the middle of those methods, ordinary Kriging and empirical Bayesian Kriging are considered the best method since it incorporates the spatial autocorrelation and it statistically optimizes the weight [54].

*Spatial scan (Sat scan) statistical analysis*. Spatial scan statistical analysis was also performed to test for the presence of statistically significant spatial clusters of HIV testing uptake using Sat Scan version 10.1 software. HIV testing uptake were taken as cases and without it as controls to fit the Bernoulli model. The numbers of cases in each location had Bernoulli distribution and the model required data for cases, controls, and geographic coordinates. For each potential cluster, a likelihood ratio test statistic and p-value was used to determine if the number of observed HIV testing uptake within the potential cluster was significantly higher than expected or not [55, 56].

### Model building for multi-level analysis

Due to clustered data nature and the within and between community variations intra-class correlation coefficient (ICC), Proportional change in variance (PCV) and MOR were computed for the sake of identifying variability among clusters. The ICC showed that there was a significant clustering effect. The MOR defined as the median value of the odds ratio between the area at the highest risk and the area at the lowest risk when randomly picking out two clusters. The PCV revealed the variation in the prevalence of HIV testing uptake among multiple sexual partners explained by factors. Bivariable analysis was done first to screen variables for multivariable analysis and those variables with P<0.2 or 0.25 in the bivariable analysis were considered for multivariable analysis. Four models will be fitted. The null model (Model null), which contains no exposure factors, will be used to assess the variability of HIV testing uptake. Individual-level factors and community-level variables will be included in the first (Model 1) and second (Model 2) multilevel models, respectively. Individual and community-level variables will be fitted simultaneously with the occurrence of HIV testing uptake in the third model (Model 3). The deviation test and the log likelihood test was used to compare models and the model with the highest log likelihood ratio and the lowest deviance was chosen as the best-fitted model. A pseudo variance inflation factor (VIF) was used to measure multi-collinearity and variables with fewer than 10 VIF values were selected. Factors with a p-value ≤ 0.2 or 0.25 will be selected as candidates for the final model. Finally, AORs with 95% CIs were calculated, and variables with p<0.05 on multivariable mixed effect analysis were considered significant predictors of HIV testing.

## Ethical clearance

Approval letter has received from DHS datasets center in order to access the data. After permission was confirmed from the international review board of Demographic and Health surveys (DHS) program data achivists to download the dataset for this study. Then data was obtained from major demographic and health survey through the online request from http://www.dhsprogram.com the investigator of this study maintained the confidentiality of the data. The confidentiality of the data was maintained anonymously and was collected according to ethical principles for medical research involving human subjects of the declaration of Helsinki.

## Result

### Sociodemographic characteristics of study participants

A total weighted 56,210 multiple sexual partners were included in this study. More than half of multiple sexual partners (32.86%) were found in the age group of 25–34 years. The majority of the study populations (53.99%) are male. Concerning wealth status of study participants around 33% are in the level of poor (Table 2).

**Table 2:** Socio-demographic characteristics of multiple sexual partners in Sub-Saharan Africa using recent DHS 2011 to 2021 GC.

Variables	Categories	Weighted frequency	Weighted percentage
**Age**	15–24	18,003	32.03
	25–34	18,473	32.86
	35–44	12,239	21.77
	45–54	6,238	11.10
	55–64	1,257	2.24
**Sex**	Male	30,346	53.99
	Female	25,864	46.01
**Marital status**	Never married	17,310	30.80
	Ever married	38,900	69.20
**Educational status**	No education	8505	15.13
	Primary level	20,193	35.92
	Secondary level and above	27,512	48.95
**Occupational status**	Not working	22,865	40.68
	Working	33,345	59.32
**Wealth status**	Poor	18,638	33.16
	Middle	11,190	19.91
	Rich	26,382	46.93
**Media exposure**	Low	8706	15.49
	High	47,504	84.51

### Sexual behavior and HIV/AIDS related factors

More than half of the study participants (53.37%) had the history of having first sex with the age below 18. Most of (75.49%) of the respondents has high level of knowledge about HIV/AIDS (Table 3).

**Table 3:** Sexual behavior and HIV/AIDS related characteristics of multiple sexual partners in Sub-Saharan Africa using recent DHS 2011 to 2021 GC.

Variables	Categories	Weighted frequency	Weighted percentage
Early sexual initiation	Yes	30,002	53.37
	No	26,208	46.63
Risky sexual behavior indicators			
Had any STI in last 12 months	Yes	3,608	6.42
	No	52,602	93.58
Had genital sore/ulcer in last 12 months	Yes	3,140	5.59
	No	53,070	94.41
Had genital discharge in last 12 months	Yes	4,657	8.29
	No	51,553	91.32
Over all	No risk	49,333	87.77
	Have risk	6,877	12.23
Level of knowledge about HIV/AIDS indicators			
Reduce risk of getting HIV: always use condoms during sex	Yes	44,408	79.00
	No	11,802	21.00
Reduce risk of getting HIV: have 1 sex partner only, who has no other partners	Yes	48,199	85.75
	No	8,011	14.25
Can get HIV from mosquito bites	Yes	12,883	22.92
	No	43,327	77.08
Can get HIV by sharing food with person who has AIDS	Yes	7,391	13.15
	No	48,819	86.85
Can get HIV by witchcraft or supernatural means	Yes	6,124	10.89
	No	50,086	89.11
A healthy looking person can have HIV	Yes	45,824	81.52
	No	10,386	18.48
Overall knowledge	Low knowledge	13,778	24.51
	High knowledge	42,432	75.49

### Community level factors

Of the total, 31,550 (56.13%) were rural residents. About 31,139(55.40%) of them belonged to the eastern Africa sub region. Majority (56.11%) of the surveys were conducted before 2014 (Table 4).

**Table 4:** Community level characteristics of multiple sexual partners in Sub-Saharan Africa using recent DHS 2011 to 2021 GC.

Variables	Categories	Weighted frequency	Weighted percentage
**Residence**	Urban	24,660	43.87
	Rural	31,550	56.13
**Survey year**	2011–2014	32,166	57.23
	2015–2021	24,044	42.77
**Sub regions in SSA**	Eastern Africa	31,139	55.40
	Central Africa	9,548	16.99
	Western Africa	12,528	22.29
	Southern Africa	2,995	5.33
**Country income level**	Low income	41,591	73.99
	Low middle income	9121	16.23
	Upper-middle income	5498	9.78

### The prevalence of HIV/ ADIS testing among multiple sexual partners in SSA

**Fig 1:**
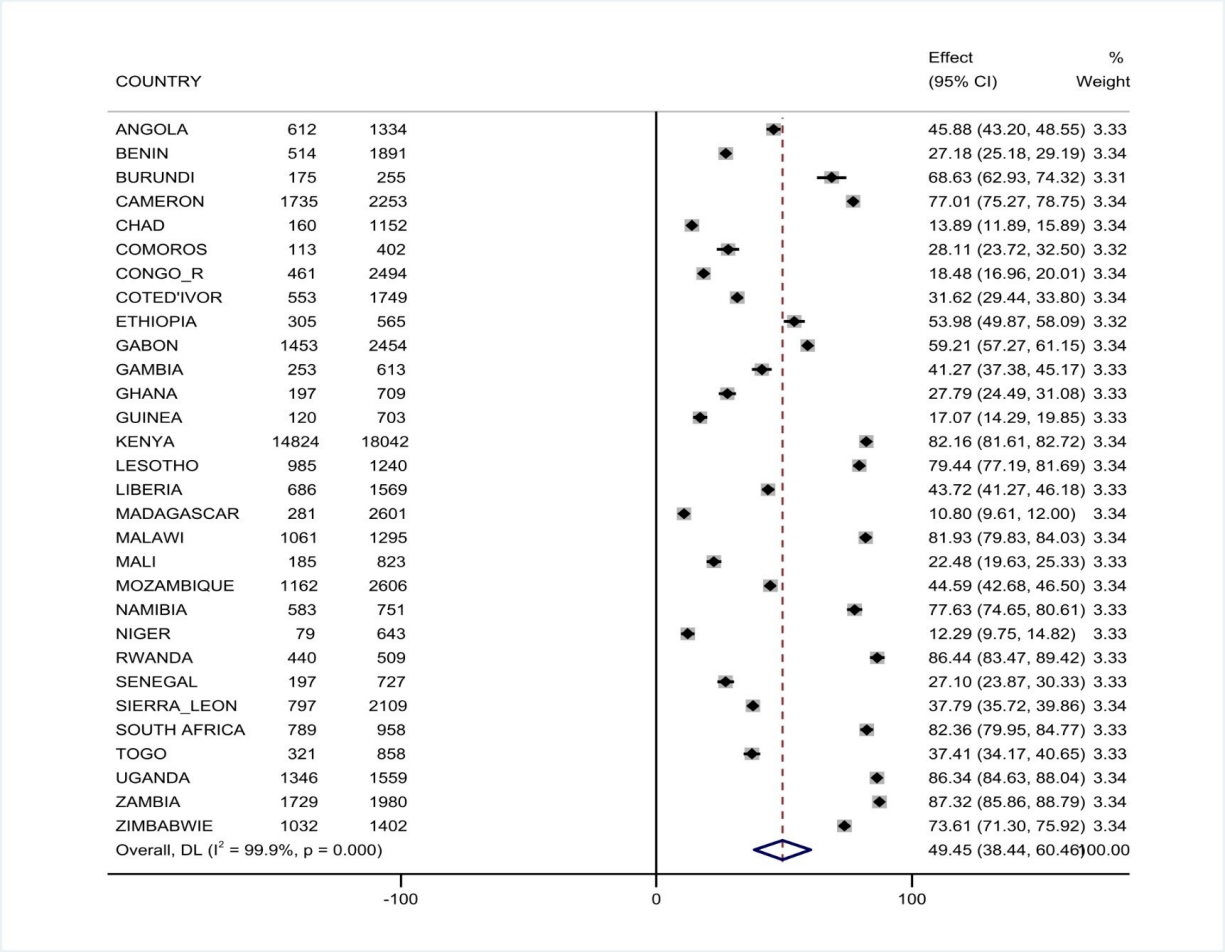
Forest plot showing the pooled prevalence of HIV testing uptake among multiple sexual partners in Sub- Saharan Africa, DHS 2011–2021.

The pooled prevalence of HIV testing among multiple sexual partners in Sub- Saharan Africa is 49.45% 95%CI (38.44%, 60.46%) with I^2^ = 99.9% and ranges from 10. 8% in Madagascar to 87.32% in Zambia (**Fig 1**).

**Fig 2:**
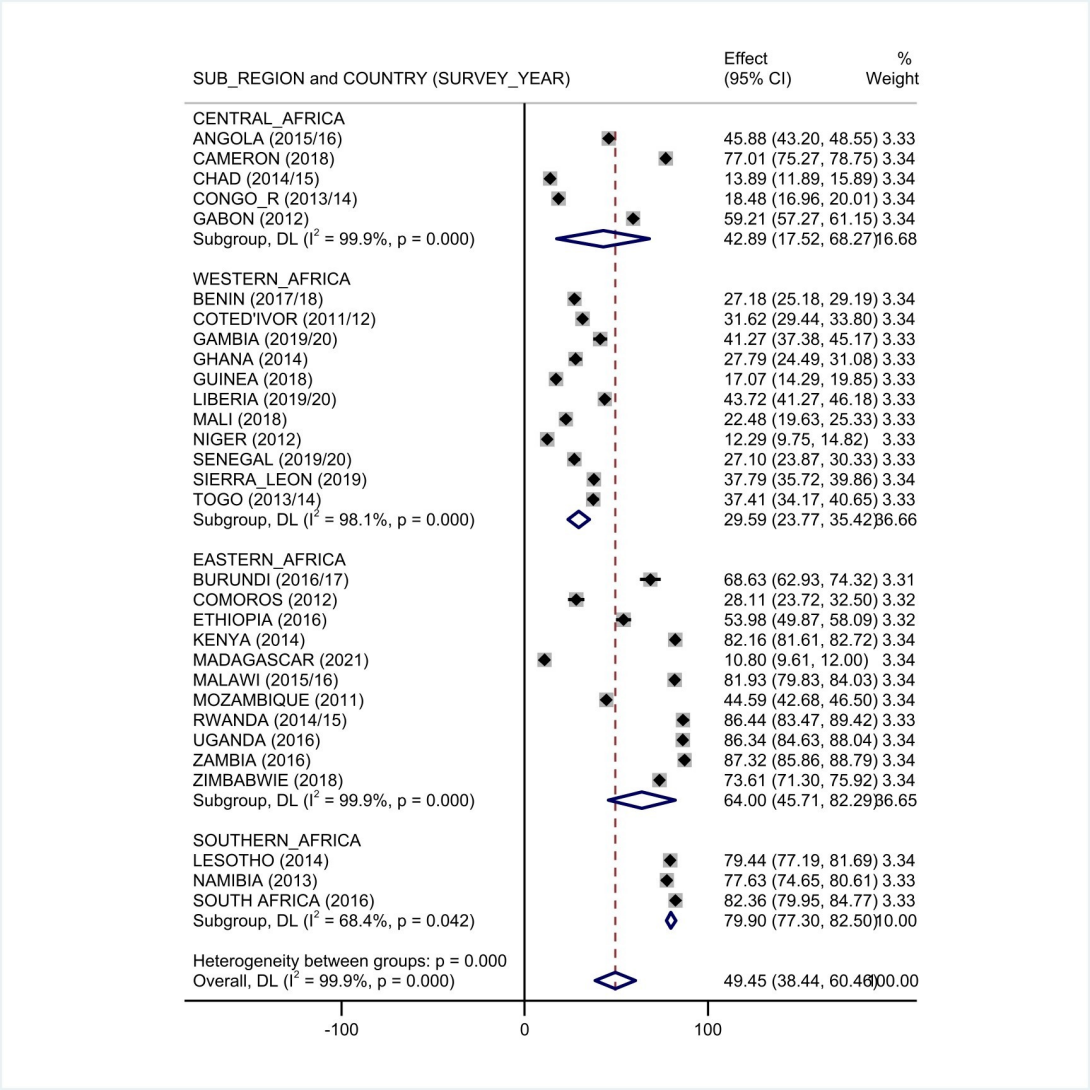
Forest plot showing the pooled prevalence of HIV testing uptake among multiple sexual partners according to the sub regions of Sub-Saharan Africa, DHS 2011–2021.

In order to obtain the pooled prevalence of each sub regions of sub Saharan African and the year in which the study conducted, further sub-group analysis was performed based on the Survey year. Based on the sub group analysis, the prevalence of HIV testing ranges from 29.59% (23.77%, 35.42%) in western African region that consists of 11 countries to 79.90% (77.30%, 82.50%) in southern region across 3 countries (**Fig 2**).

Moreover, the pooled prevalence of HIV testing according to DHS survey year conducted before and in 2014 was 45% (95% CI: 27.0,64.0) where as in the surveys which are conducted after 2014 was 52% (95%CI: 37.0,66.0).

Regarding to the country income level, the pooled proportion of uptake of HIV testing among low income countries 47% (95% CI: 31.41, 62.60), lower middle income countries 46.55% (95% CI: 24.15, 68.95) and upper middle income 66.26 (95% CI: 50.44, 82.09).

Among 30 Sub Saharan Africa, countries (Niger, Chad, Congo democratic republic, Madagascar) were found among the lowest estimates and Rwanda, South Africa, Uganda, Zambia, Kenya, Malawi from the highest estimates has a distant estimates from the pooled prevalence.

### Spatial analysis

#### Spatial distribution of low HIV testing in Sub Saharan Africa

**Fig 3:**
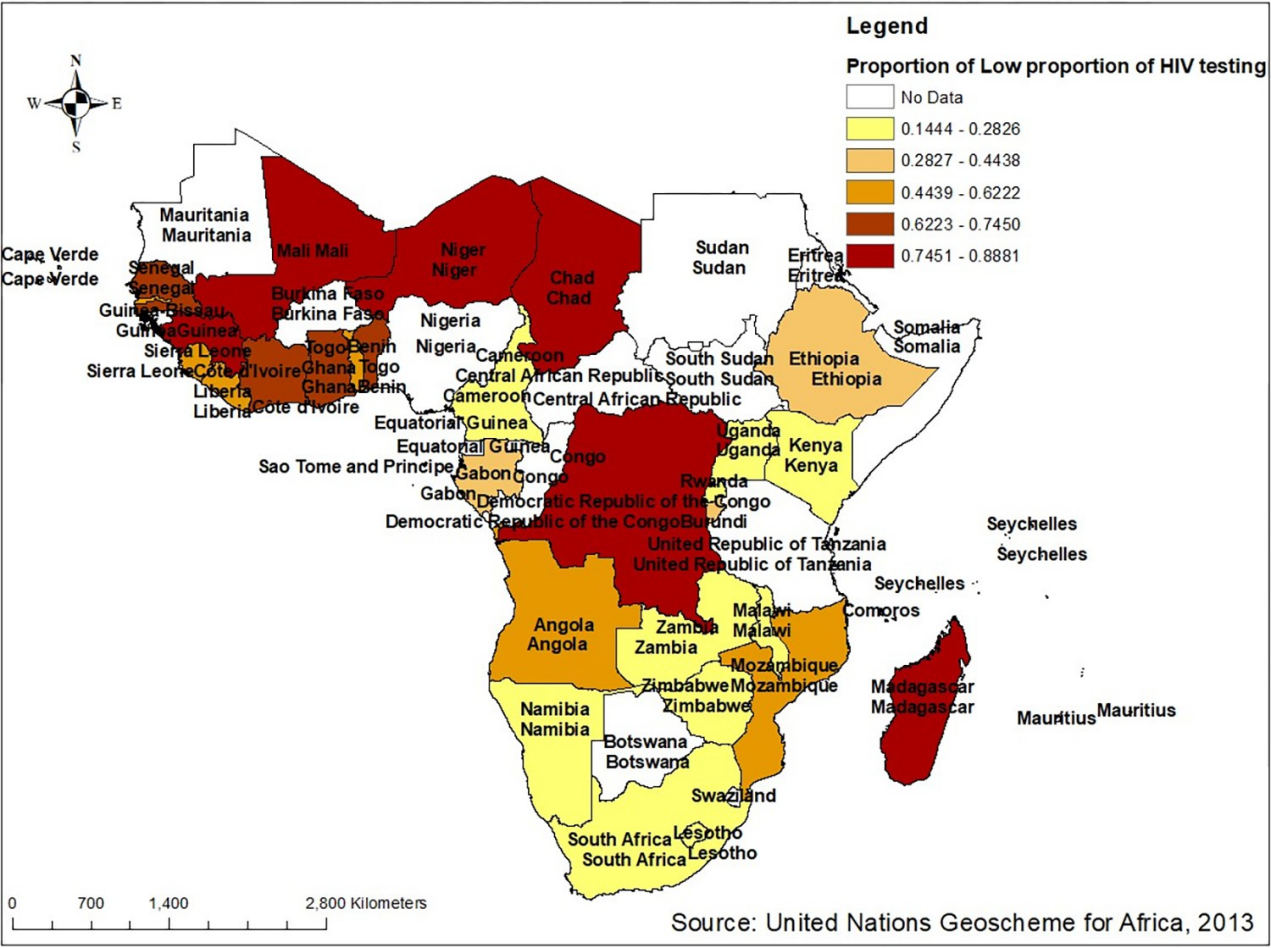
Spatial distribution of low proportion of HIV testing among multiple sexual partners in Sub- Saharan Africa based on 2011–2021 DHS. Source: United Nations geoscheme for Africa, 2013.

A total of 12,752 clusters were considered for spatial analysis of HIV testing among multiple sexual partners. The proportion of HIV testing is different through the country in sub Saharan region. The dark brown color indicates a proportion with low proportion of HIV testing almost more than 74.5% are not tested for HIV/AIDS whereas the light yellowish color indicates a high proportion of HIV testing, the proportion who are not tested is less than 28.3%. A low HIV testing proportion occurred in countries (Mali, Guinea, Chad, Niger, Congo Democratic Republic, Madagascar) found in central and western regions of sub- Saharan Africa southwest and whereas a high proportion of HIV/testing were aggregated with those countries found in the eastern and southern regions namely Uganda, Kenya, Malawi, Zambia, Zimbabwe, Namibia and South Africa (**Fig 3**).

#### Spatial autocorrelation of low proportion of HIV testing in Sub-Saharan Africa

**Fig 4:**
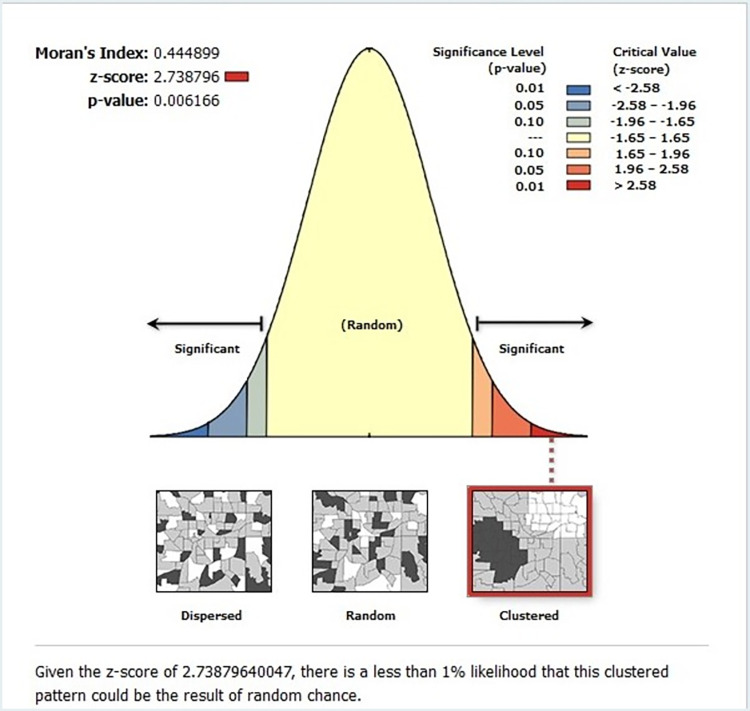
Spatial autocorrelation report of low proportion of HIV testing among multiple sexual partners in Sub- Saharan Africa based on 2011–2021 DHS.

The analysis of spatial autocorrelation indicated that the spatial distribution of low HIV testing was nonrandom in Sub-Saharan countries. The Global Moran’s I value 0.444 (p-value—0.00616) indicated that there was significant spatial autocorrelation of low HIV testing and clustering in the study area (**Fig 4**).

#### Cluster and outlier analysis of low proportion of HIV testing in Sub-Saharan Africa

**Fig 5:**
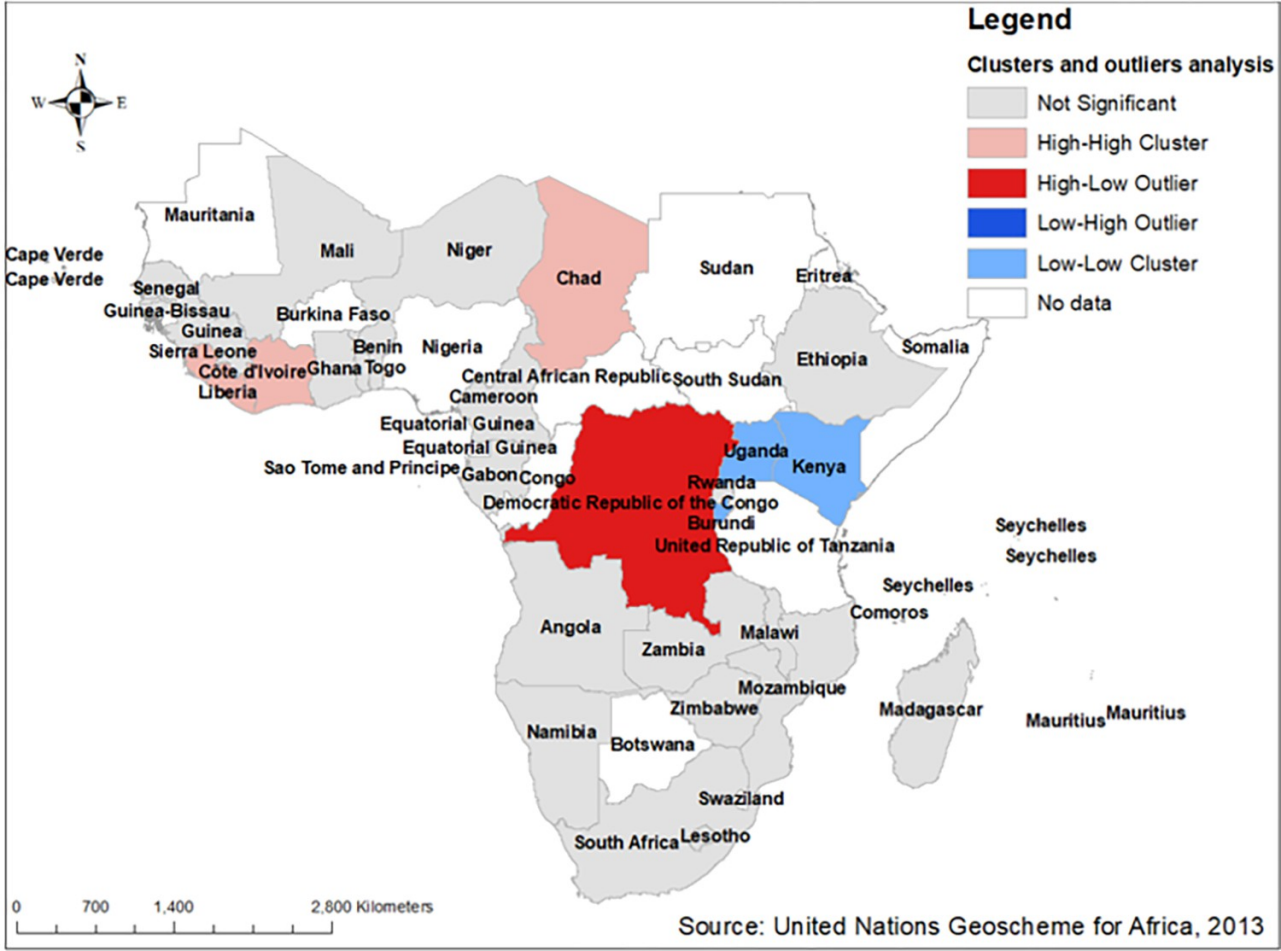
Cluster and outlier analysis of low proportion of HIV testing among multiple sexual partners in Sub- Saharan Africa based on 2011–2021 DHS. Source: United Nations geoscheme for Africa, 2013.

According to the cluster and outlier analysis, those countries found in Eastern sub-regions namely Kenya, Uganda and Rwanda were found to have low–low locations and some countries found in central and western sub regions of sub-Saharan Africa such as Chad, Cotedi’voire and Liberia had high–high locations. Generally, those areas with high-high or low-low locations that shows positive local spatial autocorrelation are referred to as spatial clusters, while Democratic Republic of Congo was the only area with high–low locations in the other hand there is no area with low–high locations. Therefore, the areas with those locations were found to have negative local spatial autocorrelation and called as spatial outliers (**Fig 5**).

#### Hot and cold spot analysis of low proportion of HIV testing in Sub-Saharan Africa

**Fig 6:**
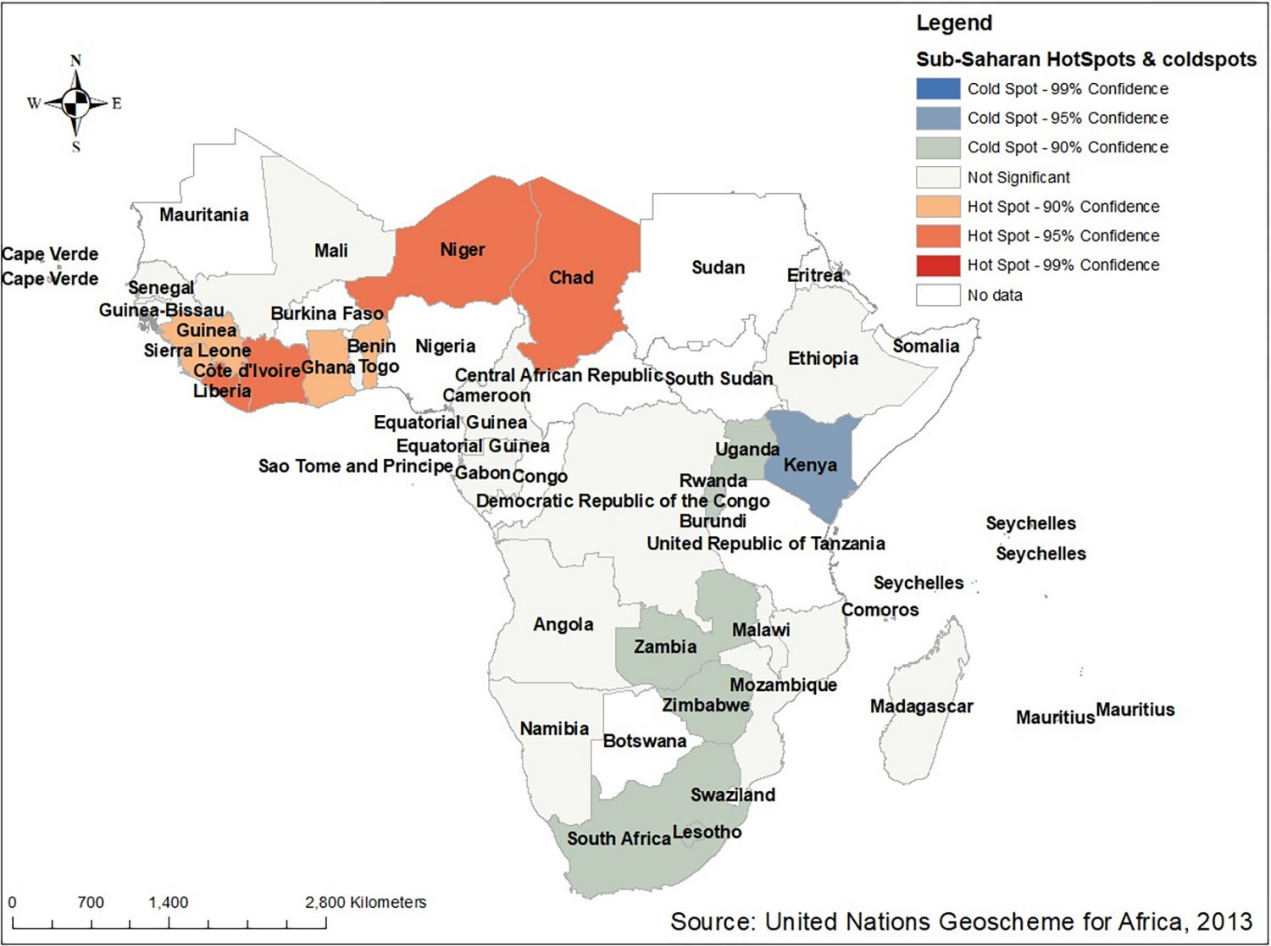
Hot spot analysis of low proportion of HIV testing among multiple sexual partners in Sub- Saharan Africa based on 2011–2021 DHS. Source: United Nations geoscheme for Africa, 2013.

In the Getis OrdGi statistical analysis, significant hotspot areas (high risk of low HIV testing) were aggregated with some part of central and western sub-region contain Niger, Chad, Benin, Ghana, Cotedi’voire, Liberia, Sera Leone and Guinea while the cold spot areas (low-risk of low proportion of HIV testing) were found in the countries of southern and eastern Sub- region (South Africa, Lesotho, Zimbabwe, Zambia, Rwanda, Malawi, Kenya, and Uganda). However most part of Sub-Saharan region such as Mali, Congo Democratic Republic, Ethiopia, Mozambique, Malawi, Angola, Namibia, Cameroon, Gabon, Senegal, and Madagascar were found to be insignificant (**Fig 6**).

#### Spatial interpolation of low proportion of HIV testing in Sub-Saharan Africa

**Fig 7:**
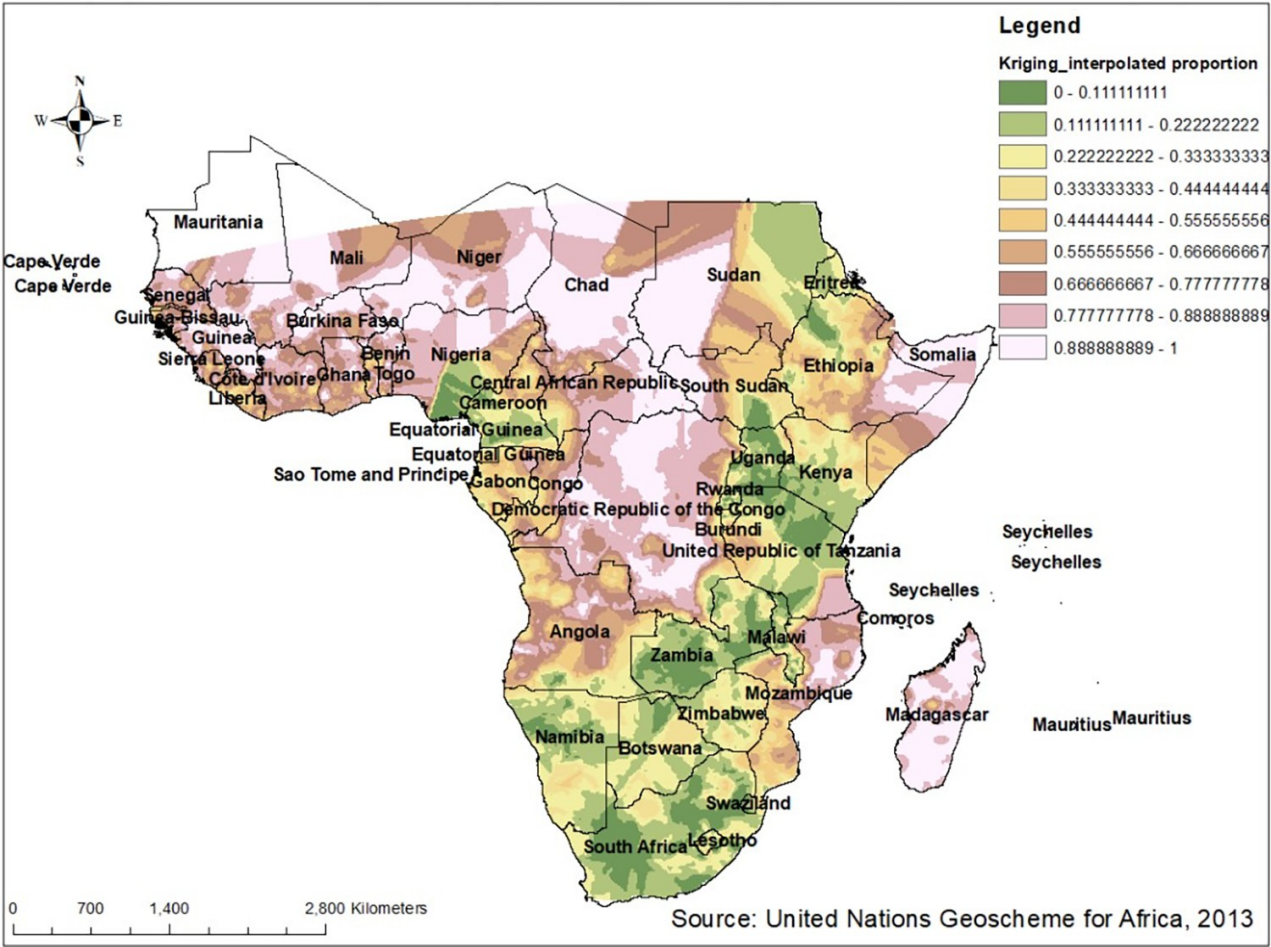
Ordinal kriging interpolation analysis of low proportion of HIV testing among multiple sexual partners in Sub- Saharan Africa based on 2011–2021 DHS. Source: United Nations geoscheme for Africa, 2013.

The ordinal kriging interpolation analysis predicted high-risk regions for low proportion of HIV testing uptake. Predication of high-risk areas was indicated by reddish color predictions, almost all part of western sub region (countries such as Benin, Cotedi’voire, Gambia, Guinea, some part of Liberia, Mali, Niger, Senegal, Sera Leone, Togo), majority part of countries in central sub region (namely Chad, Democratic Congo, Gabon and some part of Angola) and some part of eastern region of Sub-Saharan Africa that include Somalia, Comoros and Madagascar were predicted as riskier areas compared to other regions. To the opposite of this, almost all part of southern sub-region (South Africa, Botswana, Lesotho, Namibia, Eswatinia), majority of eastern sub-region countries (such as northern part of Sudan and Ethiopia, Eritrea, southern Kenya, Zambia, Malawi, Uganda, Rwanda, United republic of Tanzania) and few part of Central sub region (Cameroon and equatorial Guinea) were predicated as having the least risk for low proportion of HIV testing uptake (**Fig 7**).

#### SaT Scan analysis of low proportion of HIV testing in Sub-Saharan Africa

According to this study, about 6 significant windows that consists 6,763 clusters were identified. Of these 5744 clusters were primary clusters that were located at 8.270000 **N**, 2.700000 **E** with a 3130.42 km radius in the western and central sub- region countries such as Chad, Niger, Mali, Senegal, Guinea, Sera Leone, Cotedi’voire, Liberia, Burkina Faso, Benin, Togo, Ghana, Gabon, and some part of Democratic Republic Congo. This spatial window contained 21,310 populations located, with a relative risk of 2.23 and the Log-Likelihood Ratio (LLR) of 3185.27, at p-value < 0.001. It indicated that multiple sexual partners found inside the window were 2.23 times riskier for HIV testing utilization as compared with those found outside this widow.

The secondary significant window involved 917 clusters that were located at 20.180000 **S,** 48.220000 **E** with 1379.35 Km radius around some part of Mozambique and Madagascar. This window built in 3837 population with relative risk and the Log-Likelihood Ratio (LLR) of 2.25 and 1682.69 at p-value < 0.001 respectively.

**Fig 8:**
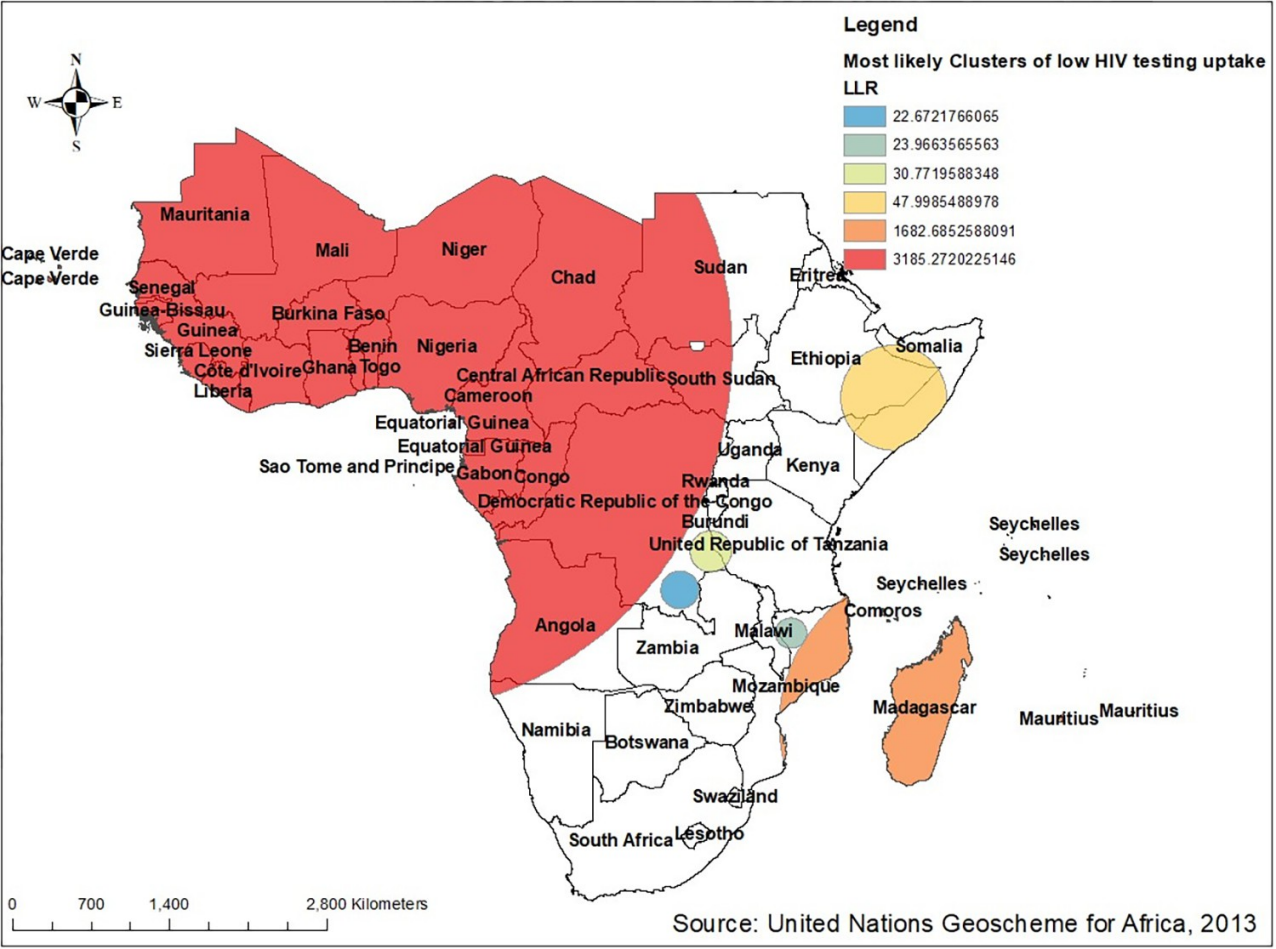
SaT Scan analysis of low proportion of HIV testing among multiple sexual partners in Sub- Saharan Africa based on 2011–2021 DHS. Source: United Nations geoscheme for Africa, 2013.

Some part of eastern sub- regions of Sub-Saharan Africa (around Southern part of Ethiopia and Somalia) was found to be the tertiary window with 52 significant clusters traced at 5.5900 **N,** 44.1800**E** with radius of 469.93 Km. In this window, around 214 populations were included with 1.81 relative risk and 47.9985 Log-Likelihood Ratio (LLR) at p-value < 0.001. The remaining three significant clusters with relative risk of 2.39, 1.74 and 2.00 at p-value < 0.001 showed as follows. (**Fig 8**).

### Factors associated with HIV testing among multiple sexual partners

#### Random effects and model comparison

The ICC in the null model indicated that 54.0% of the total variability for HIV testing uptake was due to differences between clusters/communities. Regarding PCV, about 49.47% of the variability in HIV testing uptake was explained by the full model (model III). The highest PCV in model III indicates including the community-level variables has improved the model. The median odds ratio also revealed that HIV testing uptake among multiple sexual partners was heterogeneous among clusters. In the null model, MOR was found 3.76, this means if we randomly select households from different clusters, households at the cluster with higher HIV testing uptake had 3.76 times higher odds of HIV testing uptake as compared with those households at cluster with lower HIV testing uptake. It was decreased from 3.76 in the null model to 1.90 in the final model (model III), which indicates the model III explains the low variability of HIV testing uptake (Table 5).

**Table 5:** Model comparison and parameter measurement of multi-level regression analysis.

Parameters	Null Model	Model I	Model II	Model III
Variance	3.76	2.55	2.20	1.90
ICC	53.3%	43.71%	40.07%	36.59%
MOR	6.31	4.56	4.09	3.70
PCV	Reference	32.18	41.49	49.47
Model fitness				
Log like-hood	-31276.679	-28,012.005	-29,402.797	-26,606.437
Deviance	62,553.4	56,024.01	58,805.59	53,212.87
VIF	———	1.64	1.69	1.80

ICC = Inter cluster correlation coefficients, MOR = Median odds ratio, PCV = Proportional change in variance

#### Multivariable multi-level binary logistic regression analysis

In the multilevel logistic regression analysis, both individual-level and community level-level were significantly associated with HIV/AIDS testing uptake. Among variables included in multivariable analysis sex, age, educational status, marital status, wealth index, occupation status, Media exposure, level of HIV related knowledge, residence, sub- regions in Sub-Saharan Africa, Survey year, and country income level were statistically significantly associated variables.

The odds of HIV testing uptake among multiple sexual partners who were ever married was (AOR 2.86; 95% CI; 2.66, 3.07) times higher compared to never married. The odds of HIV testing uptake among who attended primary, secondary and above education was (AOR 2.33; 95% CI; 2.13, 2.54), and (AOR 4.53; 95% CI; 4.13, 4.97) times higher compared to those who did not attend formal education respectively.

The odds of HIV testing uptake among middle and rich was (AOR 1.15; 95% CI; 1.06, 1.24), and (AOR 1.36; 95% CI; 1.25.1.47) times higher as compared to poor respectively. Similarly, being aged 25–34 years, 35–44 years, 45–54 years and 55–64 years (AOR 2.38; 95% CI; 2.21, 2.55), (AOR 2.13; 95% CI; 1.96, 2.31),(AOR 1.44; 95% CI; 1.30, 1.59) and (AOR 1.43; 95% CI; 1.19, 1.73) had positive association with HIV testing uptake. However, being male (AOR 0.29; 95% CI; 0.27, 0.31) was found to be negatively associated with HIV testing uptake as compared with those who are females.

Moreover, the study revealed that the odds of HTC uptake was higher among the countries with low middle and upper middle income level (AOR 2.41; 95% CI; 2.19, 2.72) and (AOR 2.44; 95% CI; 2.01, 2.87) compared to those living in communities with low income (Table 6).

**Table 6:** Multilevel analysis factors associated with HIV testing uptake among multiple sexual partners in Sub-Saharan Africa based on 2011 to 2021 DHS.

Variables	Categories	HIV testing status		Null model	Model I AOR (95% CI)	Model II AOR (95% CI)	Model III AOR (95% CI)
		Tested N (%)	Not tested N (%)				
**INDIVIDUAL LEVEL FACTORS**							
Age	15–24	8455(14.7)	9984(17.37)		1		1
	25–34	6083(10.58)	12878(22.41)		2.45(2.28,2.62)		2.38(2.21, 2.55)***
	35–44	4519(7.86)	7974(13.87)	-	2.08(1.91,2.25)		2.13(1.96,2.31) ***
	45–54	3109(5.40)	3214(5.59)		1.33(1.20,1.47)		1.44(1.30, 1.59)***
	55–64	846(1.47)	413(0.72)		1.15(0.95,1.39)		1.43(1.19, 1.73)***
Sex	Female	6358(11.06)	19,929(34.67)		1		1
	Male	16,653(28.97)	14,535(25.29)		0.27(0.25,0.29)		0.29(0.27,0.31) ***
Marital status	Never married	8350(14.53)	9460(16.46)		1		1
	Ever married	14,662(25.51)	25,003(43.5)		2.71(2.52,2.91)		2.86(2.66,3.07)***
Educational status	No Education	6153(10.71)	2599(4.52)		1		1
	Primary level	7423(12.92)	12998(22.62)		3.40 (3.12,3.71)		2.33(2.13,2.54) ***
	Secondary level and above	8433(14.67)	14,139(24.60)		6.29 (5.73,6.87)		4.53(4.13,4.97)***
Occupational status	Not-working	5,648(9.83)	17,288(30.08)		1		1
	Working	17,363(30.21)	17,175(29.88)		0.76(0.71,0.83)		0.88(0.81,0.95)**
Wealth index	Poor	9402(16.36)	9602(16.70)		1		1
	Middle	4835(8.41)	6489(11.29)	-	1.19 (1.11,1.29)		1.15(1.06,1.24) ***
	Rich	8775(15.27)	18,373(31.97)		1.59(1.47,1.71)		1.36(1.25.1.47)***
Risky sexual behavior	No	20,072(34.92)	30,903(53.77)		1		1
	Yes	2,940(5.12)	3561(6.20)		1.02(0.94,1.10)		1.06(0.99,1.15)
Level of knowledge about HIV/AIDS	Low	14,508(25.24)	23,986(41.73)		1		1
	High	8503(14.79)	10,478(18.23)		1.71(1.62,1.83)		1.81(1.70,1.92)***
Media	Low	3751(6.67)	4955(8.81)		1		1
	High	30,084(53.52)	17,420(30.99)		1.43(1.33,1.55)		1.43(1.32,1.55)***
**COMMUNITY LEVEL FACTORS**							
Residence	Urban	8259(14.37)	17,144(29.83)			1	1
	Rural	14,753(25.67)	17,320(30.13)			0.34 (0.32,0.37)	0.54(0.49,0.59)***
Sub-Region	Central Africa	4,950(8.61)	4,598(8.00)			1	1
	Eastern Africa	5,976(10.40)	22,565(39.26)			9.07 (7.93,10.37)	7.27(6.35,8.33) ***
	Southern Africa	2940(5.12)	2468(4.29)			8.17 (6.75,9.88)	9.87(8.16,11.94) ***
	Western Africa	9145(15.91)	4832(8.41)			0.72 (0.63,0.83)	0.85(0.74,0.98)*
Country income level	Low income	24,66(43.89)	16,923 (30.1)			1	1
	Low-middle income	5,568 (9.90)	3,553 (6.32)			2.07(1.85,2.31)	2.41 (2.19,2.72)***
	Upper-middle income	3599 (6.40)	1,899 (3.38)			2.22(1.86,2.65)	2.44 (2.01,2.87)***
Survey year	2011–2014	10,175(17.70)	22,075(38.41)			1	1
	2015–2021	12,836(22.33)	12,388(21.55)			1.05(0.97,1.14)	1.60(1.47,1.74)***

AOR = Adjusted odds ratio, COR = crude odds ratio, CI = confidence interval

* = P-value<0.05

** = P-value<0.01

*** = P-value<0.001

## Discussion

One of the key risk factors that help to explain Sub-Saharan’s high HIV prevalence is having risky sexual behaviors. Individuals with those behaviors such as multiple sexual partnership (MSP) have vital role playing in high rate of acquiring and wide spreading of HIV pandemic [57]. In order to obtain suitable response to end the pandemic, HIV/AIDS prevention strategies similar to HIV testing are crucial mainly for those vulnerable groups [46, 58]. Therefore, the present study designed to investigate the pooled prevalence, spatial distribution and associated factors of HIV testing uptake among multiple sexual partners in Sub-Saharan Africa.

Overall results revealed that around 49.5% of pooled prevalence for uptake of HIV testing among individuals with multiple sexual partnership. According to multilevel logistic regression analysis, both individual-level and community level factors were significantly associated with HIV testing uptake among multiple sexual partners in Sub-Saharan Africa. Therefore, HIV testing is improving as indicated by higher odds in countries with increased level of income, in Eastern and Southern part and with survey year of 2015–2021 than in the previous surveys. This finding applies to both males and females and then females were found to be tested better than the males. Furthermore, the more the individual be from better status of wealth, education, urbanization, the better chance to be tested.

As the study revealed that the pooled estimate of HIV testing uptake in multiple sexual partners across 30 Sub-Saharan Africa countries was 49.45%(95%CI 38.4,60.5) which is in line with the study conducted in China (52.2%) [59] and Portugal(43.5%) [18]. However, this finding is higher than another study conducted in China (12.4%) [14, 15], Germany (15.5%) [17] and those countries in Sub-Saharan Africa namely Ghana and Ethiopia (12% up to 42.2%) [60, 61]. In the other hand, it is lower than the study conducted in Georgia (66.9%) [16] and in South Africa (82.5%) [62]. This may attribute to participant socio demographic back ground, health policies and facilitation difference.

The pooled prevalence of HIV testing uptake shows discrepancy according to sub-regions of Sub-Saharan Africa. The pooled estimates of HIV testing uptake were highest in southern region of Sub-Saharan Africa with 79.9% 95%CI [77.3, 82.5] and lowest in western Africa of 29.6% with 95%CI [23.8, 35.4]. The pooled HIV testing uptake estimates in the Central region is in line, in the western region is lower and in both eastern and southern regions are higher as compared to the pooled of whole Sub-Saharan Africa. This discrepancy among regions can be explained by geographical and socioeconomic variations among the countries in each regions.

This study showed that spatial variation of HIV testing uptake in Sub-Saharan Africa was found to be varied across the countries. It identified some parts of central and western regions with high risk (low HIV testing uptake). The reason for this can be connected to regional variability in culture and religion. This may also be related to the fact that, given that the majority of HIV intervention programs are donor-driven, the success of implementing various HIV-related programs, including the expansion of HIV testing uptake, greatly depends on the ability of local implementers to use resources inefficiently and to implement interventions in the context of their communities [63, 64]. Furthermore, these discrepancies might be accounted for by the substantial geographical variation in educational status, media exposure culture, and living conditions [65].

The individual’s age was found to be a significant predictor of HIV testing uptake. This study found higher odds of HIV testing uptake among multiple sexual partners with the age over 24 years old as compared to their youngers who are those between the ages of 15–24 years old. The effect of age on HIV testing behavior observed was consistent with other studies done in Germany [17], East African countries [66], Uganda [67], Nairobi [68], Burkina Faso [63]and Ethiopia [61]. The possible reason can be that older people have had a variety of life experiences that increase their chance of contracting HIV/AIDS. However, young people consider their own risk of contracting HIV as being lower. An increased likelihood of receiving an HIV diagnosis is associated with a sense of vulnerability to ADIS. In addition to these situations, older people are more likely to engage in sexual activity and marriage than younger people, who are less likely to do so. Moreover, studies have indicated that elderly people visit health care facilities more frequently than younger people, this may allow them to use HIV testing services more effectively [69].

According to this study, being male reduced the odds of HIV testing uptake than those who are females. Several studies conducted in different areas reported similar results to those found here with reference to differences in HIV testing between sexes. For instance, studies conducted in Nairobi [68], Burkina Faso [63] and Ethiopia [61] are comparable to the results found here, showing a significantly higher uptake of HIV testing for females than males. This may be because maternity and child health care services, family planning, and other health-related services are more likely to be accessed by females. The discrepancy may therefore be the result of an increase in testing uptake during antenatal care, which will be supported by WHO recommendations for universal HIV testing of all pregnant women and for prompt treatment of HIV-positive women in order to prevent mother-to-child HIV transmission [70]. Therefore, the government and WHO could play a more positive role for all people not only for women, to better utilize HIV testing through such kinds of counseling and testing promotions.

However, this finding is argued with a previous study conducted in Australia [71] which showed that being male was strongly correlated with HIV testing uptake. This disagreement might be due to the differences in the study population.

Regarding to the marital status, the result of this study found that being ever married were associated with higher odds of HIV testing as compared to being never married. This conclusion has also been noted in previous researches carried out from Tajikistan [72], Myanmar [73], Cambodia [73], some East African countries [66] and Ethiopia [61] in Zimbabwe [10] and Nairobi [68]. The most plausible explanation for this pattern is that women who have never been married believe they have a lesser risk of contracting HIV and/or ADIS, thus they don’t think it’s necessary to get tested for the virus. Additionally, persons who have ever been married may have had the chance to get tested for HIV both during their marriage and as a partner in various medical treatments like an ANC visit [65].

Several studies have found significantly higher odds of HIV testing uptake among individuals with higher educational attainment when it is compared with those who have lower educational level. This can be supported by study which was done among Germany [17], East African countries [66], Uganda [67], Burkina Faso [63], Nigeria [74] and Ethiopia [21]. The results presented here further confirm that the odds of having been tested for HIV were significantly higher among multiple sexual partners with secondary or higher educational attainment. This finding creates discrepancies in HIV testing by education that may be brought about by variances in people’s understanding of the value of testing services, the development of health-related Information, and accessibility to testing facilities. Higher educated people may have easier access to health care services, particularly those that concentrate on how HIV/AIDS infections happen and preventative strategies. Generally, the better educated a person is, in general, the more likely they are to be accepting of and independent enough to choose how to use different healthcare facilities [75, 76].

Considering the current analysis, Employment has been significantly associated with HIV testing service uptake. This result of this study is in line with the studies of Myanmar and Cambodia, that showed the employed women were less likely to get tested for HIV than those who responded unemployed [73]. This can be due to the working pattern and schedule arrangement of the study participants. Therefore, women in any type of employment may have difficulties to have HIV test during their working hours. However, inconsistent with the study conducted in Philippines [73]. This can be explained by which being in working status aids to have income that helps to get the possibilities of affording the cost associated with HIV testing uptake (e.g., transportation to reach the HIV testing facility).

Wealth was found to be statistically significant factor to HIV testing uptake in this study. The results observed here show increased odds of having been tested for HIV among the wealthier (middle and rich) than the poorer individuals. This finding is consistent with previous studies from similar settings in East African countries [66], Nigeria [74], Ethiopia [61] and Burkina Faso [63] which found level of HIV testing increasing with wealth. Several hypotheses may explain this connotation. This may be explained by having better chance of utilizing of HIV testing services. Those who are wealthy (especially men) may be more qualified than those who are impoverished to use HIV testing. Another indication of the gap may be the fact that programs and promotions for HIV testing are not succeeding in reaching the most underprivileged groups. Additionally, the unpleasant living conditions linked to low socioeconomic status themselves could be a barrier to receiving services for HIV testing [62, 67].

Having some HIV knowledge was found a strong predictor for receiving an HIV test. The result was consistent with a previous study in Ethiopia [61], which found that individual from communities with high HIV-related knowledge led to an increased HIV testing uptake. The more that individual has better knowledge related to HIV/AIDS may get the chance of getting experiences and learning from others on the importance of uptake of HIV testing services and how they can be accessed [77].

The individual with exposure to messages about HIV testing and counseling or campaigns about condom use or abstinence can be a vital factor in motivating individuals to adopt HIV testing. Several studies conducted in Sub- Saharan countries such as Zimbabwe [10] have found positive associations between HIV testing and higher media exposure. This study found similar results and suggests that being individual who is exposed to media (i.e. listening to radio daily, watching and reading newspapers frequently) increased the odds of having been tested for HIV. This may indicate that people who are exposed to mass media may have the chance to learn about the advantages of HIV testing, as well as the locations and methods used for these services. As a result, their awareness of HIV-related information and other related concepts [78].

It should be noted that living in the rural area has protective effect on the uptake of HIV testing. Based on this study, those who are living in rural area had 45% lower odds to be tested compared with their counter parts. This finding is supported by studies conducted in East African countries’ [66] and Zimbabwe [10] referred that the participants with lower chance of testing for HIV were rural dwellers [10]. This might be evidenced due to the gap in health care services availabilities and accessibilities, infrastructural supplement problems, low level of awareness [66].

According to this study, those individuals in the countries whose survey was conducted in 2015–2021 had 1.60 times more likely to be tested when they are compared with their counterparts. The possible explanation is that the universal strategy which was announced by UNADIS might lead to the improvement of policies such as Ya Tsie Botswana Combination Prevention Project (BCPP), Sustainable East Africa Research in Community Health (SEARCH), Treatment as prevention (TasP) and other similar trails through taking it as benchmark for the monitoring and evaluation of implementation progress and outcomes related to HIV care in different portion of Sub-Saharan Africa [79–82].

Findings from the this study indicated that being from countries in Eastern and Southern Sub-regions is more likely to utilize HIV testing services compared to the central sub regions. This can be explained as both Sub-regions are trying to expand services in to community settings and improve their policy implementations that focused on the HIV/AIDS through outstanding progress to bring the region in a better position related to the minimizing proportion of epidemics and achieve the universal target of 90-90-90 alongside developed nations, since they are most affected regions by HIV/AIDS [83].

Considering the evidence that increased income level of countries in Sub-Saharan Africa has been linked to better HIV testing uptake. Those individuals in countries with the lower and upper middle income level had more likelihood to be tested than those who are in the countries of low income level. The possible explanation is perhaps individuals from countries with improved level of income may get the chance of getting highly facilitated Health care services including HIV testing services and other contributors such as infrastructures, transportations and even good budget for the HIV testing uptake campaigns [84, 85].

## Conclusion

Generally from this study, the pooled prevalence of the HIV testing uptake among multiple sexual partners in Sub-Saharan Africa region was found to be lower than the expected proportion of HIV testing services utilization by UNAIDS strategic frame work of 95-95-95.

There are significant spatial variations in the uptake of HIV testing in Sub-Saharan Africa (SSA), with hot spots (high risk of low HIV testing uptake) concentrated in western and central Africa such as Mali, Niger, Chad, Togo, Ghana, Cotedi’voire, Liberia and Madagascar.

Both individual and community- level factors affected HIV testing uptake among multiple sexual partners in Sub-Saharan Africa. Individuals with multiple sexual partnerships who were aged above 24 years, ever married, those attended education, those from rich socio-economic status, those who have comprehensive HIV related knowledge, exposure to media, those who are from the countries with Eastern and Southern sub regions, those who are from the countries with lower and upper middle income level, and those who are from the countries with survey year after 2014 were positive association with HIV testing uptake. However, being male, living in rural area and being in working status founded in western sub–region of Sub Saharan Africa were negatively associated with HIV testing uptake.

Therefore, it is recommended to give emphasis on couple-oriented HIV counselling and testing services and health education programs that focus on the creation of awareness for better HIV testing uptake. Those concerned organization such as Ministry of health, world health organization should conduct further assessment and develop policies focus on predisposing and enabling factors and also for considering males in different recommendations of HIV care in order to facilitate their utilization of voluntary testing and counseling.

## Strength and limitation

The study considered a nationally representative surveys with household and individual response of multiple sexual partners in countries of Sub-Saharan Africa therefore, results from the current analysis may be generalized to Sub Saharan Africa region as whole. Furthermore, this study measured both individual-level and community-level factors by apply multilevel analysis to reflect the hierarchical nature of the DHS data in estimating the associated factors with a combination of spatial analysis that clues an understanding of the geographic variation of HIV testing uptake.

Although the above strengths can be mentioned with the findings of this study, it has some limitations. Firstly, it counted in only multiple sexual partners, thus the findings are not generalizable to all individuals. The surveys used in the study were conducted in different years, and the status of HIV testing uptake in some of the countries may have changed and there were large heterogeneity. The information in the survey was self-reported, so to some extent under-reporting of socially unacceptable behaviors and attitudes (such as stigma), over reporting of socially desirable behaviors and recall bias were likely. Also, for some of the external variables such as health-care service factors obtained from World Health Organization Global Health Observatory Data, and other previous literatures, the most recent available data used in this study did not correspond with the DHS survey year.

## List of abbreviations

EA: Enumeration area
EDHS: Ethiopian Demographic and Health Survey
HIV: Human immune virus
ICC: : Intra-class Correlation coefficient
ICF: International Classification Function
IR: Individual women’s records
MOR: Median Odds Ratio
MR: Individual Male records
MSP: Multiple sexual partnership
PCV: Proportion Change in Variance
PLHIV: People living with HIV
STI: Sexual Transmitted Illness
SSA: Sub Saharan Africa
UNAIDS: Joint United Nations Program
VCT: Voluntary counseling and testing
VIF: Variance Inflation Factor
WHO: World Health Organization

## References

[1] Mah T.L. and Maughan-Brown B., Social and cultural contexts of concurrency in a township in Cape Town, South Africa. Culture, health & sexuality, 2013. 15(2): p. 135–147. 10.1080/13691058.2012.745951 23181362

[2] Mutinta G., Multiple sexual partnerships and their underlying risk influences at the University of KwaZulu-Natal. Journal of Human Ecology, 2014. 46(2): p. 147–155.

[3] Onoya D., et al., Determinants of multiple sexual partnerships in South Africa. Journal of Public Health, 2015. 37(1): p. 97–106. doi: 10.1093/pubmed/fdu010 24639477

[4] Amirkhanian Y.A., et al., High-risk sexual behavior, HIV/STD prevalence, and risk predictors in the social networks of young Roma (Gypsy) men in Bulgaria. Journal of immigrant and minority health, 2013. 15: p. 172–181. doi: 10.1007/s10903-012-9596-4 22370730 PMC4107306

[5] Finer L.B., Darroch J.E., and Singh S., Sexual partnership patterns as a behavioral risk factor for sexually transmitted diseases. Family planning perspectives, 1999: p. 228–236. 10723647

[6] Deeks S., et al., HIV infection. vol. 1. Nat. Rev. Dis. Primers, 2015: p. 15035. doi: 10.1038/nrdp.2015.35 27188527

[7] Amuche N.J., Emmanuel E.I., and Innocent N.E., HIV/AIDS in sub-Saharan Africa: current status, challenges and prospects. 2017.

[8] HIV/ADIS, J.o.U.N.o. Fact sheet 2022. 2022; Available from: https://www.unaids.org/en/resources/documents/2022/UNAIDS_FactSheet.

[9] Wang H., et al., Estimates of global, regional, and national incidence, prevalence, and mortality of HIV, 1980–2015: the Global Burden of Disease Study 2015. The lancet HIV, 2016. 3(8): p. e361–e387. doi: 10.1016/S2352-3018(16)30087-X 27470028 PMC5056319

[10] Magadi M.A. and Gazimbi M.M., A multilevel analysis of the determinants of HIV testing in Zimbabwe: Evidence from the demographic and health surveys. HIV/AIDS research and treatment: open journal, 2017. 4(1).

[11] Girardi E., Sabin C.A., and Monforte A.d.A., Late diagnosis of HIV infection: epidemiological features, consequences and strategies to encourage earlier testing. JAIDS Journal of Acquired Immune Deficiency Syndromes, 2007. 46: p. S3–S8. doi: 10.1097/01.qai.0000286597.57066.2b 17713423

[12] Organization W.H., Fact sheet to the WHO consolidated guidelines on HIV testing services. 2015, World Health Organization. 26378328

[13] (WHO), w.h.o. 2021; Available from: https://www.hiv.gov/hiv-basics/overview/data-and-trends/global-statistics/.

[14] Rou K., et al., Demographic and behavioral factors associated with HIV testing in China. Journal of acquired immune deficiency syndromes (1999), 2009. 50(4): p. 432. doi: 10.1097/QAI.0b013e3181946088 19322039 PMC2740799

[15] Yang Z., et al., Analysis of Multiple Sexual Partners among 2665 Male College Students Who Have Sexual Behaviour in Zhejiang Province, China. BioMed Research International, 2022. 2022. doi: 10.1155/2022/8006537 36033568 PMC9410797

[16] Tsereteli N., et al., HIV testing uptake among female sex workers and men who have sex with men in T bilisi, G eorgia. Hiv Medicine, 2013. 14: p. 29–32. doi: 10.1111/hiv.12065 24033900

[17] Kuehne A., et al., Impact of HIV knowledge and stigma on the uptake of HIV testing–Results from a community-based participatory research survey among migrants from sub-Saharan Africa in Germany. Plos one, 2018. 13(4): p. e0194244. doi: 10.1371/journal.pone.0194244 29641527 PMC5894987

[18] Dias S., et al., Patterns of sexual risk behavior, HIV infection, and use of health services among Sub-Saharan African migrants in Portugal. The Journal of Sex Research, 2020. 57(7): p. 906–913. doi: 10.1080/00224499.2019.1601154 31002270

[19] De Allegri M., et al., Factors affecting the uptake of HIV testing among men: a mixed-methods study in rural Burkina Faso. PLoS One, 2015. 10(7): p. e0130216. doi: 10.1371/journal.pone.0130216 26132114 PMC4488464

[20] Mandiwa C. and Namondwe B., Uptake and correlates of HIV testing among men in Malawi: evidence from a national population–based household survey. BMC health services research, 2019. 19: p. 1–8. 30922321 10.1186/s12913-019-4031-3 PMC6440107

[21] Ending A., progress towards the 90–90–90 targets–global AIDS update. Geneva: Joint United Nations Programme on HIV. AIDS, 2017.

[22] Data, S.M.U., UNAIDS. Đường link: http://www.unaids.org/sites/default/files/media_asset/unaids-data-2018_en.pdf, 2018.

[23] Staveteig S., et al., Reaching the ‘first 90’: Gaps in coverage of HIV testing among people living with HIV in 16 African countries. PloS one, 2017. 12(10): p. e0186316. doi: 10.1371/journal.pone.0186316 29023510 PMC5638499

[24] Ajayi A.I., et al., Concerns about contracting HIV, knowing partners’ HIV sero-status and discussion of HIV/STI with sexual partners as determinants of uptake of HIV testing. Journal of Biosocial Science, 2019. 51(4): p. 549–561. doi: 10.1017/S0021932018000330 30516121

[25] Musheke M., et al., A systematic review of qualitative findings on factors enabling and deterring uptake of HIV testing in Sub-Saharan Africa. BMC public health, 2013. 13(1): p. 1–16. doi: 10.1186/1471-2458-13-220 23497196 PMC3610106

[26] Prestage G., Brown G., and Keen P., Barriers to HIV testing among Australian gay men. Sexual Health, 2012. 9(5): p. 453–458. doi: 10.1071/SH12033 23380195

[27] JUNPo H. and HIV A., aids JUNPo: 90–90–90: an ambitious treatment target to help end the AIDS epidemic. Geneva: UNAIDS, 2014.

[28] Jacob N., et al., Consolidating strategic information to monitor progress against the UNAIDS 90–90–90 targets: evaluating the operational feasibility of an electronic HIV testing register in Cape Town, South Africa. BMC Health Services Research, 2020. 20: p. 1–14. doi: 10.1186/s12913-020-05517-7 32762660 PMC7409395

[29] Nygren-Krug H., The Joint United Nations Programme on HIV/AIDS. 2018: Oxford Scholarship Online Oxford, UK.

[30] Le Blanc D., Towards integration at last? The sustainable development goals as a network of targets. Sustainable Development, 2015. 23(3): p. 176–187.

[31] Branson B.M., et al., Revised recommendations for HIV testing of adults, adolescents, and pregnant women in health-care settings. Morbidity and Mortality Weekly Report: Recommendations and Reports, 2006. 55(14): p. 1-CE–4. 16988643

[32] Chow E.P., et al., Behavioral interventions improve condom use and HIV testing uptake among female sex workers in China: a systematic review and meta-analysis. AIDS patient care and STDs, 2015. 29(8): p. 454–460. doi: 10.1089/apc.2015.0043 26217931

[33] Matovu J.K. and Makumbi F.E., Expanding access to voluntary HIV counselling and testing in sub‐Saharan Africa: alternative approaches for improving uptake, 2001–2007. Tropical Medicine & International Health, 2007. 12(11): p. 1315–1322. doi: 10.1111/j.1365-3156.2007.01923.x 17949401

[34] Iwuji C.C., et al., Universal test and treat and the HIV epidemic in rural South Africa: a phase 4, open-label, community cluster randomised trial. The lancet HIV, 2018. 5(3): p. e116–e125. doi: 10.1016/S2352-3018(17)30205-9 29199100

[35] Biesma R.G., et al., The effects of global health initiatives on country health systems: a review of the evidence from HIV/AIDS control. Health policy and planning, 2009. 24(4): p. 239–252. doi: 10.1093/heapol/czp025 19491291 PMC2699244

[36] Johnson L.F., Access to antiretroviral treatment in South Africa, 2004–2011. Southern African journal of HIV medicine, 2012. 13(1). 10.4102/sajhivmed.v18i1.694 PMC5843157 29568630

[37] Southern African H., Fixed-dose combination for adults accessing antiretroviral therapy. S Afr J HIV Med, 2013. 14(1 Suppl): p. 41–43.

[38] Giguère K., et al., Trends in knowledge of HIV status and efficiency of HIV testing services in sub-Saharan Africa, 2000–20: a modelling study using survey and HIV testing programme data. The Lancet HIV, 2021. 8(5): p. e284–e293. doi: 10.1016/S2352-3018(20)30315-5 33667411 PMC8097636

[39] Bekele Y.A. and Fekadu G.A., Factors associated with HIV testing among young females; further analysis of the 2016 Ethiopian demographic and health survey data. PLoS One, 2020. 15(2): p. e0228783. doi: 10.1371/journal.pone.0228783 32045460 PMC7012428

[40] Olakunde B.O., et al., Spatial variations in family planning demand to limit childbearing and the demand satisfied with modern methods in sub-Saharan Africa. Reproductive Health, 2022. 19(1): p. 144. doi: 10.1186/s12978-022-01451-5 35733204 PMC9215060

[41] Croft T.N., Marshall A.M., and Allen C.K., Guide to DHS Statistics. Rockville, Maryland, USA: ICF; 2018. 2018.

[42] data, W.B. 2021; Available from: https://www.google.com/url?sa=i&rct=j&q=&esrc=s&source=web&cd=&cad=rja&uact=8&ved=0CAQQw7AJahcKEwjY2-6ym9v9AhUAAAAAHQAAAAAQBQ&url=https%3A%2F%2Fdata.worldbank.org%2Fcountry%2FZG&psig=AOvVaw1GcxkN2ZRGQxU_jt6cCeqe&ust=1678876207228216)

[43] Worldometer. Subregions in Africa by population 2021; Available from: https://www.google.com/url?sa=i&rct=j&q=&esrc=s&source=web&cd=&cad=rja&uact=8&ved=0CAQQw7AJahcKEwjw8MTCgeD9AhUAAAAAHQAAAAAQAw&url=https%3A%2F%2Fwww.worldometers.info%2Fworld-population%2Fpopulation-by-africa-subregion%2F&psig=AOvVaw3kawVOck9uugC87-za0UJ0&ust=1679041053910993.

[44] Liga A.D., et al., Predictors of multiple sexual partners among men in Ethiopia: A multilevel analysis. Ethiopian Journal of Health Sciences, 2022. 32(4): p. 689–698. doi: 10.4314/ejhs.v32i4.4 35950063 PMC9341023

[45] Bank, W. List of Economy. Available from: https://data.worldbank.org/.

[46] Erena A.N., Shen G., and Lei P., Factors affecting HIV counselling and testing among Ethiopian women aged 15–49. BMC Infectious Diseases, 2019. 19(1): p. 1–12. 31864297 10.1186/s12879-019-4701-0 PMC6925845

[47] Mazengia F. and Worku A., Age at sexual initiation and factors associated with it among youths in North East Ethiopia. Ethiopian Journal of Health Development, 2009. 23(2).

[48] 0., I.G.A.a.l. 2017; Available from: http://geoportal.icpac.net/layers/geonode%3Aafr_g2014_2013_0.

[49] Tsai P.-J., et al., Spatial autocorrelation analysis of health care hotspots in Taiwan in 2006. BMC Public Health, 2009. 9: p. 1–13. 20003460 10.1186/1471-2458-9-464 PMC2799414

[50] Anselin L., Exploring spatial data with GeoDaTM: a workbook. Center for spatially integrated social science, 2005: p. 165–223.

[51] Zulu L.C., Kalipeni E., and Johannes E., Analyzing spatial clustering and the spatiotemporal nature and trends of HIV/AIDS prevalence using GIS: the case of Malawi, 1994–2010. BMC infectious diseases, 2014. 14(1): p. 1–21. doi: 10.1186/1471-2334-14-285 24886573 PMC4057596

[52] Peeters A., et al., Getis–Ord’s hot-and cold-spot statistics as a basis for multivariate spatial clustering of orchard tree data. Computers and Electronics in Agriculture, 2015. 111: p. 140–150.

[53] Geography G., Spatial Autocorrelation and Moran’s I in GIS. 2015.

[54] Bhunia G., Shit P., and Maiti R., Comparison of GIS-based interpolation methods for spatial distribution of soil organic carbon (SOC). J Saudi Soc Agric Sci, 17, 114–126. 2018.

[55] Wieczorek W.F. and Delmerico A.M., Geographic information systems. Wiley Interdisciplinary Reviews: Computational Statistics, 2009. 1(2): p. 167–186. doi: 10.1002/wics.21 20717487 PMC2921721

[56] Jacquez G.M., et al., Global, local and focused geographic clustering for case-control data with residential histories. Environmental Health, 2005. 4(1): p. 1–19. 15784151 10.1186/1476-069X-4-4 PMC1083418

[57] Kharsany A.B. and Karim Q.A., HIV infection and AIDS in sub-Saharan Africa: current status, challenges and opportunities. The open AIDS journal, 2016. 10: p. 34. doi: 10.2174/1874613601610010034 27347270 PMC4893541

[58] Liang B., et al., Trends and associated factors in the uptake of HIV testing among female sex workers in Sino-Vietnam border areas in Guangxi, China: a cross-sectional study. BMC Infectious Diseases, 2022. 22(1): p. 1–13. 35590271 10.1186/s12879-022-07459-3 PMC9118634

[59] Li J., et al., Determinants of recent HIV self-testing uptake among men who have sex with men in Jiangsu Province, China: an online cross-sectional survey. Frontiers in Public Health, 2021. 9: p. 736440. doi: 10.3389/fpubh.2021.736440 34790641 PMC8591127

[60] Manu A., et al., Risky sexual behaviours and HIV testing among young people in Ghana: evidence from the 2017/2018 Multiple Indicator Cluster Survey. Reproductive Health, 2022. 19(1): p. 1–8. 35643502 10.1186/s12978-022-01439-1 PMC9148450

[61] Alem A.Z., et al., Determinants of HIV voluntary counseling and testing: a multilevel modelling of the Ethiopian Demographic and Health Survey. BMC women’s health, 2022. 22(1): p. 1–10. 34998389 10.1186/s12905-021-01590-0 PMC8742444

[62] Jooste S., et al., Socio-economic differences in the uptake of HIV testing and associated factors in South Africa. BMC Public Health, 2021. 21(1): p. 1–10. 34445996 10.1186/s12889-021-11583-1 PMC8390264

[63] Kirakoya-Samadoulougou F., Jean K., and Maheu-Giroux M., Uptake of HIV testing in Burkina Faso: an assessment of individual and community-level determinants. BMC public health, 2017. 17(1): p. 1–11. 28532440 10.1186/s12889-017-4417-2 PMC5441086

[64] Qiao S., et al., Facilitators and barriers for HIV-testing in Zambia: A systematic review of multi-level factors. PloS one, 2018. 13(2): p. e0192327. doi: 10.1371/journal.pone.0192327 29415004 PMC5802917

[65] Bibiana N., et al., Knowledge, attitude and factors affecting voluntary HIV counseling and testing services among women of reproductive age group in an Abuja Suburb community, Nigeria. Medical Journal of Zambia, 2018. 45(1): p. 13–22.

[66] Worku M.G., Teshale A.B., and Tesema G.A., Prevalence and associated factors of hiv testing among pregnant women: A multilevel analysis using the recent Demographic and Health Survey Data from 11 East African countries. HIV/AIDS-Research and Palliative Care, 2021: p. 181–189. doi: 10.2147/HIV.S297235 33603494 PMC7886292

[67] Jude O., Nelson O., and Katagwa I., Socio-economic and demographic factors associated with never having tested for HIV among sexually active men across the four administrative regions of Uganda. BMC Public Health, 2021. 21: p. 1–13. 34923979 10.1186/s12889-021-12384-2 PMC8684685

[68] Ziraba A.K., et al., Determinants for HIV testing and counselling in Nairobi urban informal settlements. BMC public health, 2011. 11: p. 1–10. 21861898 10.1186/1471-2458-11-663 PMC3179452

[69] Nakanjako D., et al., Acceptance of routine testing for HIV among adult patients at the medical emergency unit at a national referral hospital in Kampala, Uganda. AIDS and Behavior, 2007. 11: p. 753–758. 10.1007/s10461-006-9180-9 17096199

[70] Organization W.H., Guidelines on HIV self-testing and partner notification: supplement to consolidated guidelines on HIV testing services. 2016: World Health Organization. 27977094

[71] Sawleshwarkar S., et al., Determinants of HIV testing. Sexually transmitted infections, 2011. 87(5): p. 426–432. doi: 10.1136/sti.2011.049601 21685190

[72] Kasymova S., Uptake of HIV testing among women of reproductive age in Tajikistan: An assessment of individual determinants. Central Asian Journal of Global Health, 2020. 9(1). doi: 10.5195/cajgh.2020.370 33062400 PMC7538876

[73] Myint W.W., et al., Determinants of HIV Testing Uptake among Women (aged 15–49 years) in the Philippines, Myanmar, and Cambodia. International Journal of Maternal and Child Health and AIDS, 2021. 10(2): p. 221. doi: 10.21106/ijma.525 34900391 PMC8647194

[74] Lépine A., Terris-Prestholt F., and Vickerman P., Determinants of HIV testing among Nigerian couples: a multilevel modelling approach. Health policy and planning, 2015. 30(5): p. 579–592. doi: 10.1093/heapol/czu036 24906362

[75] Latunji O. and Akinyemi O., Factors influencing health-seeking behaviour among civil servants in Ibadan, Nigeria. Annals of Ibadan postgraduate medicine, 2018. 16(1): p. 52–60. 30254559 PMC6143883

[76] Kifle D., et al., Maternal health care service seeking behaviors and associated factors among women in rural Haramaya District, Eastern Ethiopia: a triangulated community-based cross-sectional study. Reproductive health, 2017. 14(1): p. 1–11. 28086926 10.1186/s12978-016-0270-5 PMC5237279

[77] Badru T., et al., HIV comprehensive knowledge and prevalence among young adolescents in Nigeria: evidence from Akwa Ibom AIDS indicator survey, 2017. BMC Public Health, 2020. 20: p. 1–10. 31931760 10.1186/s12889-019-7890-y PMC6956480

[78] Asp G., et al., Associations between mass media exposure and birth preparedness among women in southwestern Uganda: a community-based survey. Global health action, 2014. 7(1): p. 22904. doi: 10.3402/gha.v7.22904 24433945 PMC3888909

[79] Marukutira T., et al., Comparison of knowledge of HIV status and treatment coverage between non-citizens and citizens: Botswana Combination Prevention Project (BCPP). Plos one, 2019. 14(8): p. e0221629. doi: 10.1371/journal.pone.0221629 31465494 PMC6715216

[80] Chamie G., et al., Reaching 90–90–90 in rural communities in East Africa: lessons from the sustainable East Africa research in community health trial. Current Opinion in HIV and AIDS, 2019. 14(6): p. 449. doi: 10.1097/COH.0000000000000585 31589172 PMC6798741

[81] Iwuji C.C., et al., Evaluation of the impact of immediate versus WHO recommendations-guided antiretroviral therapy initiation on HIV incidence: the ANRS 12249 TasP (Treatment as Prevention) trial in Hlabisa sub-district, KwaZulu-Natal, South Africa: study protocol for a cluster randomised controlled trial. Trials, 2013. 14(1): p. 1–15. doi: 10.1186/1745-6215-14-230 23880306 PMC3750830

[82] Sabapathy K., et al., Achieving the UNAIDS 90–90–90 targets: a comparative analysis of four large community randomised trials delivering universal testing and treatment to reduce HIV transmission in sub-Saharan Africa. BMC Public Health, 2022. 22(1): p. 2333. doi: 10.1186/s12889-022-14713-5 36514036 PMC9746009

[83] HIV/AIDS, J.U.N.P.o., Ending AIDS: Progress towards the 90-90-90 targets. Global AIDS update, 2017.

[84] Filc D., et al., Is socioeconomic status associated with utilization of health care services in a single-payer universal health care system? International journal for equity in health, 2014. 13(1): p. 1–8. 25431139 10.1186/s12939-014-0115-1 PMC4260253

[85] Nunes B.P., et al., Desigualdades socioeconômicas no acesso e qualidade da atenção nos serviços de saúde. Revista de Saúde Pública, 2014. 48: p. 968–976.26039400

